# A practice-centered intervention to increase screening for domestic violence in primary care practices

**DOI:** 10.1186/1471-2296-7-63

**Published:** 2006-10-25

**Authors:** Denise E Bonds, Shellie D Ellis, Erin Weeks, Shana L Palla, Peter Lichstein

**Affiliations:** 1 Department of Epidemiology and Prevention, Division of Public Health Sciences, and Section of General Internal Medicine, Department of Internal Medicine, Wake Forest University School of Medicine, Winston-Salem, North Carolina, USA; 2 Duke Clinical Research Institute, Duke University, Durham, North Carolina, USA; 3 Department of Biostatistics and Applied Mathematics, University of Texas M.D. Anderson Cancer Center, Houston, Texas, USA; 4 Section of General Internal Medicine, Department of Internal Medicine, Wake Forest University School of Medicine, Winston-Salem, North Carolina, USA

## Abstract

**Background:**

Interventions to change practice patterns among health care professionals have had mixed success. We tested the effectiveness of a practice centered intervention to increase screening for domestic violence in primary care practices.

**Methods:**

A multifaceted intervention was conducted among primary care practice in North Carolina. All practices designated two individuals to serve as domestic violence resources persons, underwent initial training on screening for domestic violence, and participated in 3 lunch and learn sessions. Within this framework, practices selected the screening instrument, patient educational material, and content best suited for their environment. Effectiveness was evaluated using a pre/post cross-sectional telephone survey of a random selection of female patients from each practice.

**Results:**

Seventeen practices were recruited and fifteen completed the study. Baseline screening for domestic violence was 16% with a range of 2% to 49%. An absolute increase in screening of 10% was achieved (range of increase 0 to 22%). After controlling for clustering by practice and other patient characteristics, female patients were 79% more likely to have been screened after the intervention (OR 1.79, 95% CI 1.43–2.23).

**Conclusion:**

An intervention that allowed practices to tailor certain aspects to fit their needs increased screening for domestic violence. Further studies testing this technique using other outcomes are needed.

## Background

Domestic Violence (DV) or Intimate Partner Violence is the physical, sexual, or psychological harm to another by a current or former partner or spouse [1]. Current estimates are that 5.3 million episodes of intimate partner victimization occur each year in the United States and nearly 25% of women have experienced some form of DV in their lifetime [2]. DV is associated with poor health outcomes. Women with a history of DV have a 60% higher rate of physical health problems [3] and are 4–6 times more likely to have depression [4].

Although the adverse health consequences of domestic violence have been widely documented, there is not consensus on the effectiveness of screening. While the United State Preventive Services Task Force recently found insufficient evidence to support routine screening for domestic violence [5], other physician organizations such as the American Medical Association [6], and the American College of Obstetricians and Gynecologists [7] have stated support for inclusion of screening or awareness in medical practice. When surveyed, patients also support physicians inquiring about violence in the home [8-10]. In concurrence with the practice organizations above and in support of patient findings, the Institute of Medicine published a report in 2001 calling for increased training of health care providers on family violence [11].

Interventions to increase screening for and awareness of domestic violence by health care professionals have had mixed success [12-16]. Several studies have explored the barriers to routine screening [17,18]. Lack of education and time, and fear of offending patients are frequently cited by health care providers [17,18] as barriers to routine screening. An additional barrier that may contribute to the failure of a targeted program to increase screening is the inability of the intervention to adapt to the individual characteristics of the health care practice or professional. If the educational mode or tool to be tested is too rigid to integrate with a clinic's existing routines, it may be discarded or not adopted, resulting in a failure to change. We hypothesized that a practice-centered intervention that is sensitive to the particular needs of the practice while still remaining true to the underlying principles of quality may be more successful in implementing change. To determine if a practice-centered intervention could successfully change practice patterns, we conducted a multi-faceted intervention to increase screening for domestic violence.

## Methods

Project PAAVE (Providers Asking About ViolencE) was a three year project designed to increase the rate of screening for domestic violence by primary care providers. Conducted in western North Carolina, PAAVE was a multi-modality intervention that included both standardized educational sessions and components customized to the needs of participating practices. The intervention was evaluated through a pre/post telephone survey of female patients seen within the last 12 months at the practice. The study was reviewed and approved by the Institutional Review Board at Wake Forest University School of Medicine.

### Practices

Primary care practices (defined as internal medicine, family medicine, or obstetrics and gynecology) with at least two providers located within 50 miles of Wake Forest University School of Medicine were invited to join. Both academic and community-based practices were eligible. Providers could be physicians, nurse practitioners, nurse midwives, or physician assistants. Practices agreed to send one staff member and one provider to a centralized training session and to allow these practice members to act as an on-site resource on domestic violence and local champion to increase screening (Domestic Violence Resource Persons). Primary care practices were contacted during a three month period. Several methods were used including letters, presentations at local meetings, cold calls to practice managers.

### Intervention

The intervention for this study was multi-focal, consisting of training of two local resource persons (Domestic Violence Resource Persons), provider and staff education, audit of baseline rates and feedback of those rates back to the clinic, and ongoing educational visits (lunch and learn). Within this framework, clinics were allowed to customize specific aspects as described below.

Each Domestic Violence Resource Person attended an all day training session conducted by a noted expert in the field (Dr. Elaine Alpert, MD, MPH). The training was modeled on the Massachusetts Medical Society Seminar Series on Domestic Violence and was supplemented with sessions on legal issues pertinent to the local area and successful teaching methods to facilitate in-practice training. Practices received a copy of the training material for use at their own practice. To accommodate different practice patterns, each Domestic Violence Resource Person was allowed to select the screening instrument to be used in their clinic (from 5 previously tested instruments [19-23]), the method of screening (verbal or written), the staff person responsible for screening (provider or nursing staff), the frequency of the screening, and the patient population to be screened. At a minimum, practices were asked to screen all women over the age of 18 at least once per year. Additionally, practices selected patient education material (posters and handouts from local domestic violence advocacy agencies and the Family Violence Prevention Fund) that targeted their patient population.

Following the central training, the two Domestic Violence Resource Persons conducted either a single one and a half hour or two 45 minute training sessions at their own clinic with the help of study staff. All clinic staff and providers were asked to attend the training. Further education was conducted in the form of lunch and learn sessions. Three of these visits were conducted at 6 month intervals at each practice site and were lead by a study investigator and staff person with the assistance of the two Domestic Violence Resource Persons. The first in-clinic visit was standardized across practices and introduced the support services available in the community and reported the practices baseline screening rates. The second and third visits were customized to meet the demands of the practices and included problem solving to increase screening at their practice, additional training sessions for new providers (selected primarily by the academic practices) and exploration of the role religion may play in a victim's decision to address domestic violence (this topic was added at the request of many practices who felt that religious views may be a significant barrier to seeking services for abusive situations for their patients). Both Domestic Violence Resource Persons were also invited to a half-day refresher training session held mid-way through the project. Baseline screening rates were provided to each practice at a lunch visit with the overall screening rate for all practices as a benchmark. Finally, ongoing support was provided in the form of newsletters, a website, and telephone support by study staff.

### Evaluation

The main study outcome, change in the percentage of female patients reporting screening by their health care provider in the past 12 months for violence in the home, was assessed by a telephone survey. Two cross-sectional, random samples of adult, female patients at each practice were surveyed. To be eligible for the survey, patients had to be female, over the age of 18, speak English (due to lack of bilingual staff), be able to understand and respond to questions, and have been seen in the clinic in the 12 months prior to the interview. The pre-intervention survey was conducted prior to any training session and prior to clinics explicitly stating the proposed extent of screening at their practice. Thus, the pre-intervention survey was limited to female patients, the minimal screening recommended by study staff. For consistency, the post-intervention survey was also limited to women, despite several clinics choosing to screen men in addition to women. Patients were interviewed for the baseline survey between July and September 2002. The follow-up survey of patients was conducted between August and October 2004.

The survey was conducted in a similar manner at each time point. Each clinic supplied a list of at least 400 female patients to the research team. Most practices provide a list of all female patients seen at the clinic in the twelve months prior to the survey. One clinic solicited patients for participation via a sign-up list located in the waiting room. The research team then randomly selected patients to participate using a random number table. Selected patients were contacted by telephone by the research team, verbal consent obtained, and the survey conducted. At least three attempts at different times of the day were made to contact the participant. If a selected patient was not reached after three attempts or refused, another patient was randomly selected. This sequence was continued until 100 completed surveys were obtained from each clinic at each point in time (baseline and follow-up; total from each clinic n = 200).

A previously validated survey was used for the telephone survey [24-26]. Rather than using a survey solely devoted to screening for domestic violence and potentially compromising women living in abusive situations, the outcome question was embedded in a survey on general healthcare. The survey asked participants whether their health care provider had asked about particular behaviours that may affect health, and conducted or ordered screening clinical exams. Additional questions regarding health care utilization and patient demographic characteristics were also included. The behavioural health risks included in the survey were: smoking, alcohol or drug use, physical inactivity, keeping a gun in home, and experiencing safety or violence in home, family/relationship concerns, poor sexual functioning, and stress. Patient demographic factors included age, marital status, race, income, presence of children in the home, and medical insurance. Health care utilization questions included the number of visits for any type of health care in the last year, and gender and specialty of the health care provider. Our primary outcome measure was a "Yes" response to the question "In the past 12 months has a doctor or other health professional discussed with you concerns about safety or violence in the home."

### Compensation

In recognition of the time and potential productivity loss by the Domestic Violence Resource Persons, each practice received $1,500 compensation. Five hundred dollars was provided after completion of the initial telephone survey and the remaining $1000 after completion of the follow-up survey. The stipend was paid to the practice, not to individual providers, and was not based on the change in screening rates. No compensation was provided to patients who completed the survey.

### Statistical Analysis

Analyses were performed using STATA version 8.0 (Stata Corp., College Station, TX). Descriptive statistics using means and standard deviations were used to look for patterns in the change in screening rate among practices. Chi-squared and Student's t test were used to determine differences in patient demographics between the initial and follow-up samples. The odds ratio of being screened after the intervention was determined. To account for the effect of clustering by clinic site on screening rates, generalized estimating equations were used. The unadjusted odds ratio was first calculated. We then adjusted for patient and practice characteristics. Specifically, we modelled the odds ratio of having been screened as a function of being in the follow-up sample after adjusting for age, marital status, race, income insurance, primary care provider gender and speciality, number of visits, and clustering by clinic.

## Results

Seventy-nine practices were contacted by mail or phone during the three month recruitment period. Seventeen academic (n = 5) and community-based (n = 12) primary care practices were recruited, among them 4 internal medicine, 11 family practice, and 2 obstetric and gynecology giving an overall practice response rate of 22%. Two practices are excluded from our analysis. One practice dropped out after the initial training session and one closed shortly after the study began (both community-based family practice).

### Intervention selections

All 15 practices sent at least one representative to the initial training session and 13 sent two. All practices completed the one and half hour training session at their own clinics and participated in all academic detailing sessions. Thirteen of the practices sent at least one Domestic Violence Resource Person to the mid-intervention training session and seven sent both. Ten practices had at least one of the original Domestic Violence Resource Person still at their practice at the end of the intervention.

A wide variety of screening instruments were selected. Seven practices selected the SAFE screening instrument [20]. Four selected questions from the Massachusetts Medical Society Seminars on Domestic Violence [19] while the remaining used either the Partner Violence Screen (n = 2) [21] or the HITS questionnaire (n = 2) [22]. Four practices chose to conduct the minimal screening (women over age 18), while four practices decided to screen all women regardless of age; five opted to screen both men and women over the age of 18 and three chose to screen adult and adolescent patients. Most practices chose to screen once per year (N = 12).

### Change in Screening

At baseline and follow-up, 1,482 and 1,527 women were surveyed, respectively. The mean age of women surveyed was 48 years (see Table 1). Participants were primarily white, approximately half were married, and more than a third had at least one child under the age of 18 living in the house. The majority of both samples had some form of health insurance, and about half worked outside the home. Half of both samples reported annual household income of less than $25,000. The mean number of visits to a health care provider in the last year was similar between the two groups as was the number of women who reported seeing a female primary care provider. In the follow-up sample, slightly more participants reported seeing a family medicine provider.

**Table 1:** Survey respondent characteristics

Characteristic	Initial Survey (N = 1,482)	Follow-up Survey (N = 1,527)	P value
Age (mean, SD)	49 years (SD = 17.7 years)	48 years (SD = 16.9)	NS
Race			
White	62% (894)	66% (987)	0.04
African American	37% (531)	32% (471)	
Other	2% (23)	2% (33)	
Married or marriage-like relationship	46% (683)	54% (833)	<0.0001
Child under age 18 living at home	40% (594)	41% (627)	NS
Health Insurance-Yes	90% (1,337)	89% (1,363)	NS
Working Full or Part-Time	46% (676)	50% (761)	0.05
Income less than $25,000	50% (580)	50% (661)	NS
Visits to health care provider in last year (mean, SD)	7.5 (SD = 9.3)	8.0 (SD = 11.1)	NS
Female PCP	52% (755)	48% (716)	0.02
Type of PCP			
Family Medicine	60% (831)	64% (911)	<0.0001
Internal Medicine	18% (255)	18% (261)	
OB/GYN	9% (123)	8% (111)	
Midlevel	10% (143)	6% (83)	
Other	3% (44)	3% (48)	

At follow-up a significant increase was observed in the proportion of women reporting they had been screened for safety or violence in the home (see Table 2). At baseline, 16% of the women surveyed stated they had been screened in the previous 12 months. By practice, this ranged from 2% of participants reporting being screened to 49%. Following the intervention, 26% (p < 0.0001 for the comparison to screening at baseline) of survey participants reported having been screened, with the range among practices 8% to 62%. The range of increase in screening by practice was 0% to 22%. When clustering by clinics was accounted for using GEE modelling, the odds ratio of being screened after the intervention was 1.79 (95% CI 1.45–2.20). This remained stable after adjustment for patient (age, race, marital status, work status, children in home, income, insurance status, number of visits) and practice (gender and type of PCP) characteristics (OR 1.79, 95% CI 1.43–2.23).

**Table 2:** Percent of patient reporting screening for violence in the home

	Pre-Intervention Survey (N = 1,482)	Post-Intervention Survey (N = 1,527)	Unadjusted OR (95% CI)	Adjusted** OR (95% CI)
Women reporting they had been screened in past 12 months	16% (N = 236)	26%* (N = 398)	1.79 (1.45–2.20)	1.79 (1.43–2.23)
Range of screening by practice	2% to 49%	8% to 62%		

*p < 0.001 for comparison with pre-intervention survey

**Adjusted for patient age, race (white, non-white), marital status (married, not married), insurance, income (<$25,000, >$25,001), presence of children at home, number of visits, primary care provider gender, primary care provider type (family medicine, internal medicine, OB/Gyn, midlevel provider, other)

Although the small sample size precluded tests of significance, in descriptive statistics internal medicine practices and academic practices demonstrated the largest gain in screening (see Table 3). Larger practices also had larger gains in screening. Although practices that did not send a DVRP to the mid year training had no change in screening rate, those practices that did not have a DVRP present at the end of the study had similar increase to those that had both. Practices that designated screening to be performed by provider or both nurses and providers experienced larger gains than those that designated nurses to conduct the screening.

**Table 3:** Mean increase in screening by practice characteristic and intervention choice

Practice Characteristics	Number of practices	Mean change in screening after intervention (SD)
Practice Type		
Internal Medicine	4	13% (7%)
Family Medicine	8	8% (7%)
Obstetric/Gynecology	3	10% (11%)
Academic	5	13% (6%)
Community	10	8% (8%)
Number of providers at practice		
1–3	3	7% (13%)
4–10	6	6% (4%)
11 or more	6	15% (5%)
Number of DVRPs attending initial training		
1	2	9% (15%)
2	13	10% (7%)
Number of DVRPs attending mid-project training		
0	2	0% (2%)
1	6	11% (4%)
2	7	11% (9%)
Number of original DVRPs present in practice at end of study		
0	5	11% (8%)
1	4	7% (5%)
2	6	10% (9%)
Screening questionnaire used		
SAFE Questions ^[20]^	7	9% (7%)
Massachusetts Seminars Series on Domestic Violence ^[19]^	4	11% (9%)
Partner Violence Screen ^[21]^	2	13% (9%)
HITS ^[22]^	2	5% (9%)
Person designated at practice to screen		
Nurse Staff	4	1% (2%)
Providers	9	12% (6%)
Both	2	16% (8%)
Patients to be routinely screened		
Women over age 18	4	10% (5%)
All women	4	12% (10%)
Men and Women over age 18	5	9% (8%)
All patients	2	6% (10%)
Frequency of screening		
Once a year	12	10% (7%)
At every visit	3	10% (10%)
Baseline screening rate		
<8%	3	4% (3%)
8–10%	4	11% (7%)
11–19%	4	11% (10%)
>20%	4	11% (9%)

## Discussion

Using a practice-centered intervention, we obtained a ten percent absolute increase in patient-reported screening for domestic violence. When patient characteristics, health care provider characteristics and clustering by practice were accounted for, patients were 79% more likely to have been screened after the intervention than at baseline (OR 1.79, 95% CI 1.43–2.23).

The success of this intervention may be due, in part, to the ability of the clinics to customize the intervention to fit their own clinical needs. Our goal was to increase screening within an intervention framework that had been shown to be effective in changing provider behaviour. We drew upon previous studies that have shown that provider education [27], audit and feed back [28,29], local champions [30], and educational outreach visits (lunch and learn) [31,32] can be successful in changing behaviour. Recognizing that single focus interventions are less-successful than ones that include a variety of change mechanisms [33], we devised a multi-focal framework. We further sought to increase the success of the study by allowing each clinic to customize aspects to fit their individual needs. The Domestic Violence Resources Persons at each practice worked with study staff to determine the type of screening instrument, frequency, method and patient to be screened. Throughout the study, ongoing educational sessions were tailored to fit individual clinic needs. This was perhaps most noticeable in the academic practices. Although all practices received the same number of academic detailing visits, the academic practices were more likely to choose a review of the basic techniques of screening for domestic violence as an orientation for new interns.

Although the small number of clinics participating precluded comparisons by clinic type, we did see a trend toward a larger increase in screening in larger practices, academic practices and those with higher baseline rates of screening. While we cannot determine the underlying cause for the variability in change seen, it is possible that the selection of a review of the basic techniques of screening by the academic practices served to reinforce the basic message to screen. Other factors may also have influenced the larger increases seen in academic practices. Residents may have received prior training on domestic violence during medical school and PAAVE provided a review of that training and a reinforcement of the techniques used for screening. The larger increase seen in clinics with higher baseline screening rates may reflect a greater ease in increasing a screening practice than in instituting one. This may also reflect a greater commitment to screening in some clinics.

The increase in screening seen in PAAVE is similar to that found in other studies. Thompson *et al *tested a one year intensive intervention in primary care clinics and found a 14.3% difference between intervention and control practice in screening for DV (6.2% control, 20.5% intervention [34]. A briefer intervention was performed in community clinics in the northeast which resulted in a 20% absolute increase in screening rates (5% to 25%) six months following the intervention [35]. Our intervention differs from these in the length of the intervention (eighteen months), and that we allowed practices to select aspects of the intervention based on their needs.

While we attribute the increase in screening to the intervention, we are limited somewhat by the lack of a control group. This study was not randomized and all practices received the intervention. Thus, it is possible that the increase in screening was due to a secular increase in overall rates of screening for behavioural health risks or that some influence outside of our intervention was responsible. Our outcome question, "...concerns about safety or violence in the home?" was general and may have been misinterpreted by some of the patients to refer to more generalized safety issues at home rather than domestic violence specifically. It is also possible that the increase in screening is due in part to the compensation provided to practices. We minimized the effect this would have by providing payment to the practice rather than specific providers and not making the payment contingent on the change in screening rates. Finally, the small number of practices included in the intervention precluded more controlled analyses to determine specific practice characteristics associated with increased screening.

## Conclusion

Our study demonstrates that an increased rate in screening for domestic violence can be obtained through the use of practice-centered intervention. Although this study was limited by the lack of a comparison group, it appears that the technique of customizing aspects of the intervention to better fit the specific needs of the practice can be a successful strategy for changing practice patterns. Improving quality of care in the outpatient setting remains an area of increased attention and focus. Techniques to improve care and alter provider practice are needed. Application of this technique with other health care outcomes and rigorous study design is needed. If this technique proves successful in other settings and with other interventions, it will provide a valuable addition to tools available to improve medical care.

## Competing interests

The author(s) declare that they have no competing interests.

## Authors' contributions

DEB designed the study, conducted intervention visits, and wrote the manuscript; SDE assisted with intervention design, conducted intervention visit and edited all versions of the paper; EW assisted with the intervention design, supervised data collection and edited all versions of the paper; SLP conducted data analysis and edited all versions of the manuscript; PL assisted with intervention design, conducted intervention visits to practices and edited all versions of the manuscript. All authors read and approved of the final manuscript.

## Pre-publication history

The pre-publication history for this paper can be accessed here:


